# Rapid Detection of Ebola Virus with a Reagent-Free, Point-of-Care Biosensor

**DOI:** 10.3390/s150408605

**Published:** 2015-04-14

**Authors:** Justin T. Baca, Virginia Severns, Debbie Lovato, Darren W. Branch, Richard S. Larson

**Affiliations:** 1 Department of Emergency Medicine, University of New Mexico School of Medicine, Albuquerque, NM 87131, USA; 2 Department of Pathology, University of New Mexico School of Medicine, Albuquerque, NM 87131, USA; E-Mails: VSeverns@salud.unm.edu (V.S.); DLovato@salud.unm.edu (D.L.); RLarson@salud.unm.edu (R.S.L.); 3 Sandia National Laboratories, Albuquerque, NM 87185, USA; E-Mail: dwbranc@sandia.gov

**Keywords:** pathogens, infectious diseases, viruses, piezoelectric, biosensors, diagnosis, SAW, surface acoustic wave

## Abstract

Surface acoustic wave (SAW) sensors can rapidly detect Ebola antigens at the point-of-care without the need for added reagents, sample processing, or specialized personnel. This preliminary study demonstrates SAW biosensor detection of the Ebola virus in a concentration-dependent manner. The detection limit with this methodology is below the average level of viremia detected on the first day of symptoms by PCR. We observe a log-linear sensor response for highly fragmented Ebola viral particles, with a detection limit corresponding to 1.9 × 10^4^ PFU/mL prior to virus inactivation. We predict greatly improved sensitivity for intact, infectious Ebola virus. This point-of-care methodology has the potential to detect Ebola viremia prior to symptom onset, greatly enabling infection control and rapid treatment. This biosensor platform is powered by disposable AA batteries and can be rapidly adapted to detect other emerging diseases in austere conditions.

## 1. Introduction

The 2014 Ebola virus outbreak is the largest in history, with widespread transmission in multiple West African countries and sporadic cases in Europe and North America [1,2,3,4]. The massive public health response has been limited, in part, by the inability to rapidly detect the presence of Ebola virus in potential patients living in remote areas [5]. The early symptoms of Ebola overlap with symptoms of endemic malaria and other febrile illnesses [6].

Rapid, point-of-care detection of the Ebola virus could enable early quarantine and halt future epidemics and pandemics [5,6]. While point-of-care nucleotide amplification tests exist [5], these are limited by the need for multiple reagents, refrigeration, and specialized personnel. Here, we present preliminary results of a Surface Acoustic Wave (SAW) biosensor with potential to detect Ebola virus in multiple, unprocessed sample types including blood, serum, saliva, and feces within 5–10 min.

Ebola virus is a class A select agent filovirus that was first identified in Zaire in 1976 and named after the River Ebola in Zaire [7]. Ebola outbreaks are sustained through person-to-person transmission from direct contact with infected people, bodily fluids, or contaminated clothes and linens [7]. Early detection of this highly contagious virus would allow for improved infection control measures and early treatment at specialized facilities. The current mortality rate from epidemic Ebola ranges from 40% to 90% and could be greatly reduced with early, point-of-care diagnostics [8,9]. Mathematical models suggest that the total direct costs of the present outbreak range from $82 million to $356 million in the three most effected countries; early diagnosis and treatment will also reduce aggregate healthcare costs [10].

Current approaches to Ebola identification include antigen-capture ELISA testing, IgM ELISA, RT-PCR, virus isolation, electron microscopy, and serologic testing for IgM or IgG antibodies. Quantitative measurement of the viral load also has prognostic importance, with a much higher case fatality rate in patients with viral loads over 10 million copies per milliliter [11]. Current tests for Ebola, especially PCR-based techniques, are generally limited by the need for added reagents, refrigeration, and the need for specially trained laboratory personnel. Lateral flow assays can provide rapid and fairly inexpensive qualitative results, but are unable to quantify viral load.

Our group has developed a point-of-care biosensor that employs surface acoustic waves for label-free pathogen detection without the need for any sample preparation [12,13,14]. This biosensor platform detects target antigens in the presence of confounding analytes and in various types of media, thus eliminating complicated sample preparation protocols. Such acoustic wave sensors can achieve limits of detection of <50 pg/cm^2^, which is an order of magnitude lower than detection limits for other marker-free systems such as optical surface plasmon resonance (SPR) and quartz crystal microbalance (QCM) devices [15].

With this sensor platform, viral detection is rapid (with acoustic wave signal detection within 2 min), and the entire detection protocol (from collecting the test sample to obtaining a positive or negative test result) requiring about 5–10 min. Such technological benefits would be ideal for rapid, point-of-care identification and diagnosis of the Ebola virus, especially in environments with minimal or overloaded infrastructure, such as in public health or emergency response situations. Here, we discuss initial modifications of the biosensor to demonstrate detection of Ebola and its potential utility to combat this recurrent epidemic.

## 2. Experimental Section

### 2.1. Fabrication and Functionalization of the SAW Biosensor

The sensor chips were prepared using lithium tantalate (36°, y-cut, x-propagating LiTaO_3_) wafers by lithographic deposition and patterning of inter-digital transducers (IDT) and waveguide layers as previously described [12]. Briefly, cleaned LiTaO_3_ wafers were patterned with four IDT patterns with sets of transducers and delay lines using negative tone photoresist AZ2020 (AZ Electronic Materials, Branchburg, NJ, USA). Then, a metallization step was performed with 5000 Å aluminum using an electron-beam evaporator (Temescal, Wilmington, MA, USA). The wafer was placed in an acetone bath to lift off the photoresist and any excess aluminum, followed by an acetone spray as needed and by the following rinses in methanol, isopropyl alcohol, and deionized water. This process was repeated for ground metallization, bus lines, and contact pads. Next, a waveguide layer of 5000 Å silicon dioxide (SiO_2_) was deposited as a film on the wafer using lift-off plasma enhanced chemical vapor deposition (Oerlikon Versaline, Pfaeffikon, Switzerland). The oxide was coated with hexamethyldisilazane in a vacuum oven, and positive tone photoresist AZ4330 (AZ Electronic Materials) was used to form a photoresist mask on the wafer having exposed portions. The exposed portions of SiO_2_ was etched with reactive ion etching to access electrical contact pads. Finally, the resultant wafer was diced to form individual chips, in which photoresist from dies was removed by rinsing in acetone, methanol, and isopropanol.

Antibodies were individually patterned on sensing lanes as previously described [16]. Briefly, sensor chips were coated with toluene and 3-glycidyloxypropyl trimethoxysilane (90%/10%) in an oven at 60 °C for 1.5 h and then rebaked at 100 °C for 1 h. Each lane was coated with antibodies at a concentration of 10 µg/mL in phosphate-buffered saline (PBS). The following antibodies were used: mouse monoclonal antibodies (IgG2a isotype) specific for Ebola (AB-EB-MAB1, anti-Ebola virus monoclonal antibody 1; BEI Resources, Manassas, VA, USA); and mouse IgG1 antibody isotype control (F(ab’)_2_ fragment) (ab37426, monoclonal isotype control; Abcam, Cambridge, MA, USA). Typically, two lanes were functionalized with antibodies specific for the target analyte, and two lanes were functionalized with isotype control antibodies; the latter are referred to as reference lanes.

### 2.2. Provenance and Handling of Ebola Virus Strain Zaire (Mayinga) Inactivated

The Ebola virus antigen sample (NR-31807; BEI Resources, Manassas, VA, USA) consists of inactivated and highly disrupted viral particles and was used as provided. Fully intact Ebolavirus is highly pathogenic and designated BSL-4 material. For research purposes, the supplier provides inactivated and disrupted Zaire (Mayinga) Ebola virus (BSL-1 material), which was prepared as follows. As stated by the supplier, Zaire (Mayinga) Ebola virus from infected Vero E6 pellets was suspended in 50 mM sodium borate, gamma irradiated (5 × 10^6^ rads total dose) on dry ice, and sonicated. Culture cell debris were removed by centrifugation. The virus was confirmed non-viable (killed or inactivated) by inoculation of cell culture (10 days on Vero cells) followed by a second passage (10 days on Vero cells). The concentration of the Ebola virus antigen sample was provided in plaque-forming units (PFU/mL) by the supplier, indicating the concentration of viable viral particles prior to inactivation, and allowing for comparison across different animal models and experimental conditions [17].

The Ebola antigen samples were analyzed by scanning electron microscopy at the University of New Mexico Health Sciences Center Electron Microscopy Facility (Albuquerque, NM, USA) to roughly determine the size distribution of the degraded viral particles. Ebola samples were stored in aliquots of 50 µL in screw cap safety microtubes at −80 °C until ready for use.

When ready to be tested, the samples were thawed on ice and supplemented with phosphate buffered saline (PBS; 137.0 mM NaCl, 2.7 mM KCl, 10.0 mM Na_2_HPO_4_·2H_2_O, 2.0 mM KH_2_PO_4_, pH 7.4 with HCl). All Ebola antigen samples were prepared fresh for each experiment.

### 2.3. Virus Detection Using the SAW Biosensor

Detection of Ebola Zaire antigen was conducted in BSL-2 certified biosafety cabinets. Data expressed as phase shift (Δφ, expressed in degrees) were recorded with a custom acquisition program developed using Visual Studio (Microsoft) as previously described [12]. In brief, data from each IDT delay line were collected for each lane and acquired simultaneously as a function of time of acquisition. The biosensor supports a surface shear wave, and the IDT detects the wave by transducing the mechanical wave into an electrical signal. This electrical signal (expressed as voltage information) was converted to phase (φ, expressed in degrees) using the custom acquisition program. Data from reference lanes were subtracted from the data from Ebola test lanes at each time point to determine the specific Ebola signal. The data for quantitative measurement of specific Ebola particle detection refer to the phase shift (Δφ), which corresponds to the difference between the reference and Ebola signals that is measured continuously after addition of a viral antigen sample

Ebola antigen samples of various concentrations were prepared in 100 µL of PBS and applied to the biosensor. Binding of viral antigens to the sensor surface resulted in a net phase shift of the signal Δφ (expressed in degrees on the y-axis) along the time plot (on the x-axis). While phase shift differences associated with specific Ebola signal are apparent and stable within seconds of sample addition, we continue measuring up to 5 min after sample addition to assure signal stability and reproducibility. All measurements represent multiplicates of at least three, and data points are represented as means ± standard error of the mean (SEM).

## 3. Results and Discussion

We present here the initial development and characterization of a biosensor for point-of-care, quantitative detection of the Ebola virus. This device enables log-linear, concentration-dependent detection of disrupted viral particles in the clinically relevant range. Overall sensitivity and limit of detection are dependent on the mass of the species that is bound to the sensor surface; this biosensor is likely to be orders of magnitude more sensitive for intact, infectious Ebola viral samples [15].

### 3.1. Design Elements of Surface Acoustic Wave Biosensor

The biosensor was adapted to allow for sensitive detection of the Ebola virus. The biosensor has a planar, piezoelectric substrate containing inter-digital transducers (IDTs) [12,13]*.* The piezoelectric substrate propagates horizontally polarized surface shear waves, and such waves are induced by applying an alternating voltage to the IDTs at a high frequency (generally between 80 and 400 MHz). The surface shear waves are characterized by a particular resonant frequency that is sensitive to changes on the sensor surface.

**Figure 1:**
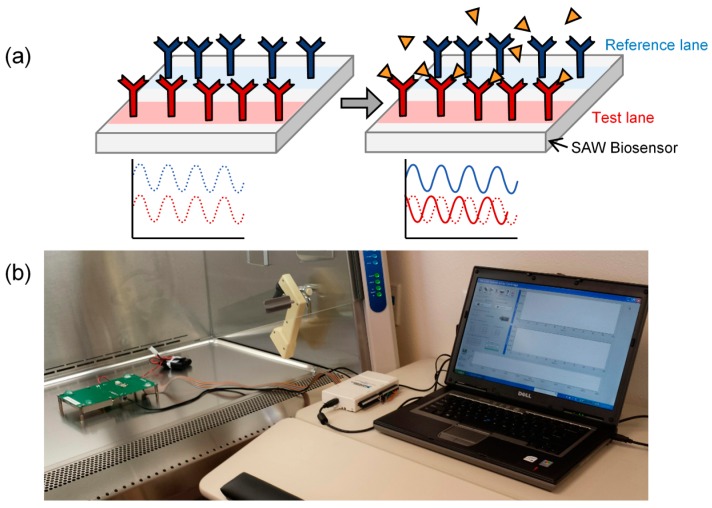
Biosensor. (**a**) Schematic of a surface acoustic wave (SAW) biosensor having a test lane functionalized with an antibody (**red**) specific for the target bioagents (triangles). The biosensor also includes a reference lane functionalized with a control antibody (**blue**); (**b**) The detection system for research use with fluidic housing surrounding the biosensor, an output interface device connected to the biosensor, and a laptop computer. The sensor is powered by disposable AA, and further miniaturization of the output interface is possible for field use. In addition, the fluidic housing and biosensor can be provided as a disposable module that can be detached from the output interface and then decontaminated prior to disposal.

Specificity for the target bioagents is obtained by functionalizing the surface of the substrate (Figure 1a). Here, the surface of the piezoelectric substrate was sensitized with a monoclonal antibody specific for Ebola virus. A binding event causes a change in surface mass and results in a phase shift of the signal wave propagating across the sensor surface. Biochemical interactions occurring on the sensor surface can be quantified by measuring this change in phase shift (Δφ).

The overall schematic concept of the biosensor device for detecting the Ebola virus is shown in Figure 1. The biosensor includes a test lane including antibodies selective for the target bioagents (*i.e.*, the Ebola virus in this study), as well as a reference lane including a control IgG1 antibody (Figure 1a). Measurements of Δφ were performed for both the test lane and the reference lane by use of an output interface device (connected to the SAW biosensor) and a laptop computer (Figure 1b).

### 3.2. Imaging Analysis of the Ebola Zaire Antigen Sample

**Figure 2:**
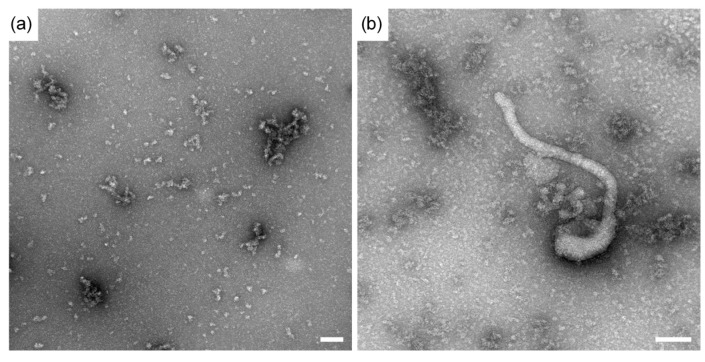
Transmission electron microscopy (TEM) images of inactivated Ebola Zaire virus sample showing predominance of fragmented viral particles. (**a**) A representative image of dilute samples containing the fragmented, inactivated Ebola virus; (**b**) An image of rare filamentous structures having a diameter of approximately 30 nm. Scale bars are 100 nm.

Fully intact Ebolavirus is highly pathogenic and designated BSL-4 material. For this particular study, we employed inactivated and disrupted Zaire (Mayinga strain) Ebola virus (BSL-1 material), which is available for research purposes under non-BSL-4 conditions. Thus, transmission electron microscopy studies were conducted to identify the size distribution of the fragmented viral particles. Representative images are provided in Figure 2. Shown is a TEM image of a 1:20 diluted sample (with PBS), which includes a majority of particles that are less than 10 nm and the presence of larger aggregates between about 50 nm to 300 nm (Figure 2a). Rare filamentous structures, with diameter of about 30 nm (Figure 2b) were also noted. Intact Ebola virus has a filamentous structure with diameter of 80 nm and length greater than 950 nm [18].

TEM of the inactivated virus sample shows highly disrupted, fragmented particles that form aggregates (Figure 2a), rather than the elongated filaments observed in intact filovirus. When filamentous structures were observed (Figure 2b), the cross-section diameter was less than half of the diameter expected for intact filovirus [18]. While the predominant species in the inactivated virus sample are disrupted particles, the biosensor phase shift response is likely to be much greater for an infectious Ebola sample containing intact virus. Based on our previous study of HIV detection [16] and the relative masses of intact HIV and Ebola [18,19], we would estimate a 5–10× greater sensitivity for intact Ebola virus compared to intact HIV. As a first approximation, we assume a log-linear correlation between mass and phase shift. As particles of greater mass will provide a greater response, the sensor is likely to detect more massive, intact viral particles at lower concentration.

### 3.3. SAW Biosensor Detection of the Ebola Zaire Sample

In analogy to our previous report on the detection of HIV-1 and HIV-2 [16], we functionalized the SAW biosensor with monoclonal antibodies specific for Zaire (Mayinga) strain of Ebola Virus. Ebola virus antigens were detected in PBS solutions over a 2.5 log reference range at concentrations corresponding to 1.0 × 10^4^ PFU/mL to 3.0 × 10^6^ PFU/mL prior to virus inactivation. The lowest concentration is below the average viremia level of 3 × 10^4^ RNA copies per mL observed in Ebola patients on the first day of disease symptoms [20].

**Figure 3:**
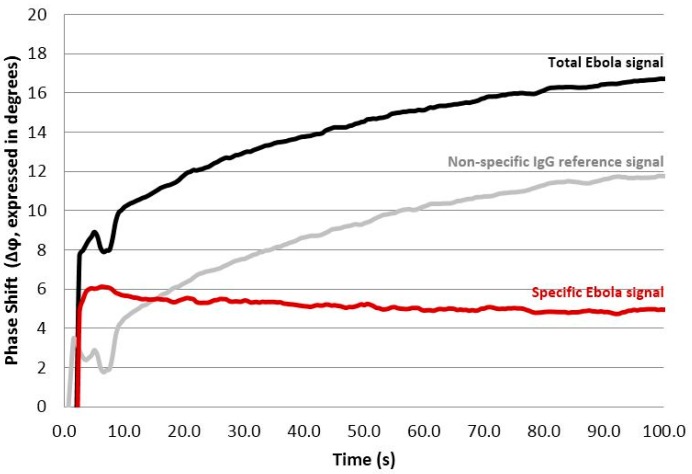
Sensor response and calculated phase shift Δφ in degrees as a function of acquisition time. Graph showing data from Ebola (**solid black line**) and reference (**gray line**) IDTs for test viral antigen with a concentration of 1,000,000 PFU/mL. The specific Ebola signal (calculated by subtracting the Non-specific IgG signal from the Total Ebola signal) is also shown (**red line**). Sample addition occurs at time 0, and several seconds are required for the signal to stabilize.

Representative phase shift data are provided in Figure 3. The IgG control lane accounts for matrix effects and non-specific binding of potentially interfering species.

**Figure 4:**
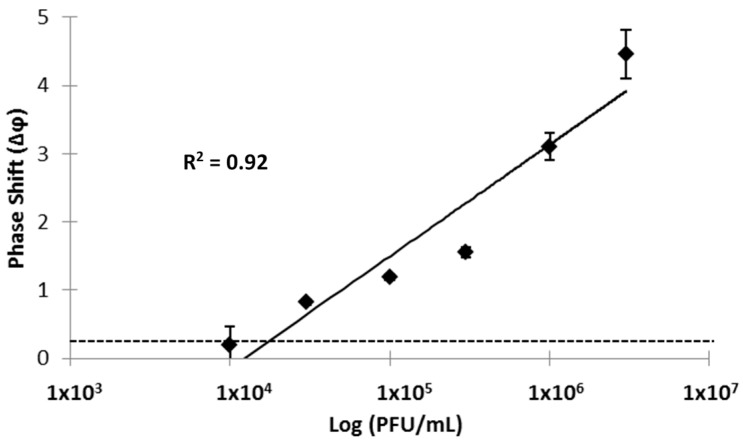
Detection of Ebola-Zaire with a SAW biosensor functionalized with an anti-Ebola monoclonal antibody. Triplicate measurements (mean ± standard deviation) were taken at 5 min after addition of the virus. Standard deviations for three of the data points are smaller than the corresponding size of the data marker. The x-axis and y-axis indicate viral load (logarithmic scale; PFU/mL) and phase shifts (Δφ, expressed in degrees), respectively. Concentrations of Ebola antigen corresponding to PFU of intact virus were established in a defined buffer (PBS). The solid line represents a linear fit and the correlation coefficient (*R*^2^) of 0.92 suggests operation in a linear range. The horizontal dotted line represents the background signal obtained with buffer without virus, and is the basis for LOD calculations.

Detection of Ebola virus resulted in a concentration-dependent increase with Δφ values ranging from 0.20 ± 0.04 to 4.46 ± 0.86, corresponding approximately to 1.6 × 10^4^ PFU/mL to 6.5 × 10^6^ PFU/mL (Figure 4). There was a log-linear relationship between viral load and Δφ for this concentration range of viral particles with a correlation coefficient *R*^2^ of 0.92. The linear range and correlation coefficients compare favorably to those recently reported for qRT-PCR [21]. A limit of detection (LOD) of 1.9 × 10^4^ PFU/mL was calculated for Ebola virus by linear regression and using the average background noise Δφ value of 0.31. These results indicate that the prototype SAW biosensor rapidly detects the Zaire strain of Ebola antigens in a defined buffer with detection limits below the average viremia level at onset of clinical symptoms.

## 4. Conclusions/Outlook

We have demonstrated an adaptable, label-free sensing system for the rapid detection of bioagents and show its use to detect the Ebola Zaire virus. We have previously shown the effectiveness of this system in detecting the *Bacillus anthracis* bacteria simulant, *Bacillus thuringiensis* [13]; the Coxsackie virus [12]; the Sin Nombre hantavirus [12]; the *Francisella tularensis* bacteria [22] and the Human Immunodeficiency Virus types 1 and 2 [16]. This study further extends the capability of this SAW biosensor platform to accommodate the rapid, label-free, and specific detection of the Ebola Zaire virus.

As fully intact Ebola virus is highly contagious, we used an inactivated virus in order to conduct this study under non-BSL-4 conditions. However, in a first responder or real world scenario, the sample would not necessarily have to be inactivated and could be tested directly, providing the sensor was contained in a controlled environment.

The SAW biosensor described here provides the basis for a rapid and specific response to Ebola outbreaks and other emerging diseases. This point-of-care sensor will provide rapid diagnosis and improved infection control, dramatically decreasing the human and economic costs of this disease. Further work will focus on portability and optimization for field use. A disposable cartridge can be employed to house the piezoelectric substrate and fluidic channels to guide the test sample to the substrate. The user interface can be readily simplified to provide a simple positive/negative result to a field worker on the front lines of the next epidemic.

Sample preparation is not required for this label-free sensing methodology, simplifying use in field conditions without centralized laboratories or refrigeration. While additional testing can be conducted to verify the detection limits for Ebola Zaire in complex media, such as blood, serum, or saliva [23], we believe that Ebola virus detection in such media will be guided by our past studies that show effective detection of other viruses in complex solutions (e.g., serum, plasma, river water, and sewage effluent [12,14,16]). In summary, the SAW biosensor is a versatile platform that shows promise to revolutionize rapid pathogen detection and enable early treatment in public health and emergency responses.
